# Evaluation of digital real-time PCR assay as a molecular diagnostic tool for single-cell analysis

**DOI:** 10.1038/s41598-018-21041-5

**Published:** 2018-02-21

**Authors:** Chia-Hao Chang, Daxen Mau-Hsu, Ke-Cheng Chen, Cheng-Wey Wei, Chiung-Ying Chiu, Tai-Horng Young

**Affiliations:** 1 Institute of Polymer Science and Engineering, National Taiwan University, Taipei, 106 Taiwan; 2 Institute of Biomedical Engineering, National Taiwan University, Taipei, 100 Taiwan; 3 National Taiwan University Hospital, Taipei, 100 Taiwan; 4 Quark Biosciences, Inc., Hsinchu County, 302 Taiwan

## Abstract

In a single-cell study, isolating and identifying single cells are essential, but these processes often require a large investment of time or money. The aim of this study was to isolate and analyse single cells using a novel platform, the PanelChip™ Analysis System, which includes 2500 microwells chip and a digital real-time polymerase chain reaction (dqPCR) assay, in comparison with a standard PCR (qPCR) assay. Through the serial dilution of a known concentration standard, namely pUC19, the accuracy and sensitivity levels of two methodologies were compared. The two systems were tested on the basis of expression levels of the genetic markers vimentin, E-cadherin, N-cadherin and GAPDH in A549 lung carcinoma cells at two known concentrations. Furthermore, the influence of a known PCR inhibitor commonly found in blood samples, heparin, was evaluated in both methodologies. Finally, mathematical models were proposed and separation method of single cells was verified; moreover, gene expression levels during epithelial–mesenchymal transition in single cells under TGFβ1 treatment were measured. The drawn conclusion is that dqPCR performed using PanelChip™ is superior to the standard qPCR in terms of sensitivity, precision, and heparin tolerance. The dqPCR assay is a potential tool for clinical diagnosis and single-cell applications.

## Introduction

Lung cancer is the most common cause of cancer-related death in the world^1^. Most of these deaths are caused by the metastasis of lung cancer tumors, leading to circulating tumor cells (CTCs) spreading cancer to other areas of the body^2–4^. Early detection of CTCs in the blood is important for the prevention of metastatic disease. Additionally, the characterization of the individual patient’s CTCs can provide clinically relevant information for physicians to diagnose and treat the patient. Accurately characterizing CTCs can potentially enable personalized medical and therapeutic decision-making for each patient. Thus, single-cell analysis can facilitate an understanding of the characteristics of an individual cell and provide more detailed information in functional genomics^5^.

Micromanipulation is one of the oldest techniques for isolating cells^6^. It entails first using a microscope to observe specific cells, then using micropipettes to aspirate the cells, and finally moving the cells to a collection vessel. This technique allows direct access to a single cell, but the process is time-consuming and inefficient, meaning it is unable to achieve high throughput. In recent years, many technologies for separating single cells have been developed^5^. Specifically, four new methods of note have been developed: fluorescence-activated cell sorting^7^, in which suspended cells are sorted at high speed by detecting droplets containing single cells that have been labelled with a fluorescent signal and light scatter characteristics; magnetic-activated cell sorting^8^, in which the suspended cells are labelled with antibody-conjugated magnetic beads, then sorted in a magnetic field; laser capture microdissection^9^, in which a laser is used to melt the thermoplastic film, allowing individual cells to adhere to the melted film to obtain the cells; and microfluidics^10–13^, in which physical, chemical, and biochemical parameters are combined to allow precise control of fluid-flow behaviour in a sub-millimetre-scale space to achieve cell separation.

Besides, owing to the rapid development of microfluidics^14^, it has become easier to obtain and analyse single-cell samples.

Fluidigm launched a C1 system, which was the first instrument to automate the division of a cell sample into single cells for genomics analysis. A disposable microfluidics device (C1IFC) is the key to its ability to capture individual cells, and it allows the execution of single-cell lysis processes in a chamber. After reverse transcription and preamplification, the preamplified products can be harvested for further analysis and application. A C1IFC can handle up to 96 single cells per hour^15^. Illumina Bio-Rad developed a single-cell sequencing platform, which is integrated with a droplet generation microfluidics technique and a sequencing system. Suspension cells are partitioned into single cells and coencapsulated with barcoded beads in subnanolitre droplets. Lysed cells’ mRNA is released and bound to the barcoded beads for synthesizing the first strands in a droplet. RNA-seq libraries are subsequently prepared and sequenced using a sequencing system^16^.

However, one of the challenges in these technologies is needing several complex processes for isolating, purifying, and analysing genomic functions of single cells. Herein, we propose a new device combining a microfluidic approach and a digital PCR technique to rapidly distribute single cells in the arrays of nano-litre-scale-well-based chips and real-time analysing genomic functions of distributed single cells.

Digital PCR (dPCR) was first described in the 1990s as a method to supersede qPCR in terms of sensitivity and reliability^17^, and many studies have supported this claim^18,19^. The process of dPCR is, for the most part, the same as that of qPCR; the key difference is that the nucleic acid sample is diluted and separated into numerous individual partitions that are independently amplified^20,21^. The copy number of nucleic acids is then calculated using Poisson statistics on the number of positive versus negative partitions^22^. Physically separating the sample into small partitions allows for a more effective PCR amplification^23^ and increased PCR inhibitor resistance^24,25^.

When sampled at very low concentrations, the target molecule is no longer in a normal distribution and instead in a Poisson distribution in solution, resulting in larger sample deviations^26^. Therefore, in the analysis of low-concentration samples, the number of replicates must be increased to provide sufficient statistical significance.

Moreover, dPCR technology based on the principle of ‘dilution’ and ‘partition’ is advantageous with a low-concentration sample. The use of a larger input sample volume reduces the sampling error and can accurately determine the sample concentration. The fact that the sample is physically separated into small partitions allows for a better PCR amplification^23^ and an increased PCR inhibitor resistance^24,25^.

PanelChip™ Analysis System (Quark Biosciences) including the instrument (PanelStation™), the microwells chip (PanelChip™), the operating software, analysis algorithm and all the essential components or accessories. The system is a digital real-time PCR assay and was created out of the need to have both the advantages of partitioning a diluted sample and the ability to monitor the amplification of DNA in real time. In addition, the Poisson distribution can be applied to disperse cells^27^. The cells are scattered on thousands of individual partitions with different distributions. At an appropriate cell concentration, each partition contains either one cell (single cell) or zero cells (empty). In theory, as long as the number of loading cells is controlled and qPCR is utilised, obtaining the signal generated from single cells is relatively easy.

To confirm the performance of dqPCR, a pUC19 plasmid DNA standard and total RNA extracts from A549 lung carcinoma cells were used, and the levels of GAPDH, E-cadherin, N-cadherin, and vimentin at various concentrations were quantified using both qPCR and dqPCR. Furthermore, the concentration of GAPDH at various concentrations of the PCR inhibitor heparin was measured.

After the preceding experiments were performed, a probability statistics model of the cell distribution across the system was created using the Poisson distribution to calculate the probability of single- and multiple-cell-containing wells. Subsequently, an experiment was conducted to determine whether the probabilities generated by our model would agree with practical results. The genes of E-cadherin, vimentin, and N-cadherin in A549 lung carcinoma cells were selected as the objects of the experiment. The cells were treated with TGFβ1 to induce epithelial–mesenchymal transformation^28^, after which they were divided into single cells. The expression levels of the three mRNA molecules in cells without the drug treatment were then compared 1 day, 2 days, and 4 days after the drug treatment.

We concluded that the new device proposed in this article is suitable to general protocol of PCR in laboratory level and with a powerful potential for clinical application and make the use of single-cell research easier.

## Results

### Performance of dqPCR

To compare performance against a conventional qPCR assay, a dqPCR was achieved using the PanelChip™ Analysis System (Quark Biosciences), and it includes thermal control, real-time fluorescence detection, and a microwell chip.

#### Serial dilution of pUC19 plasmid DNA standard

**Figure 1:**
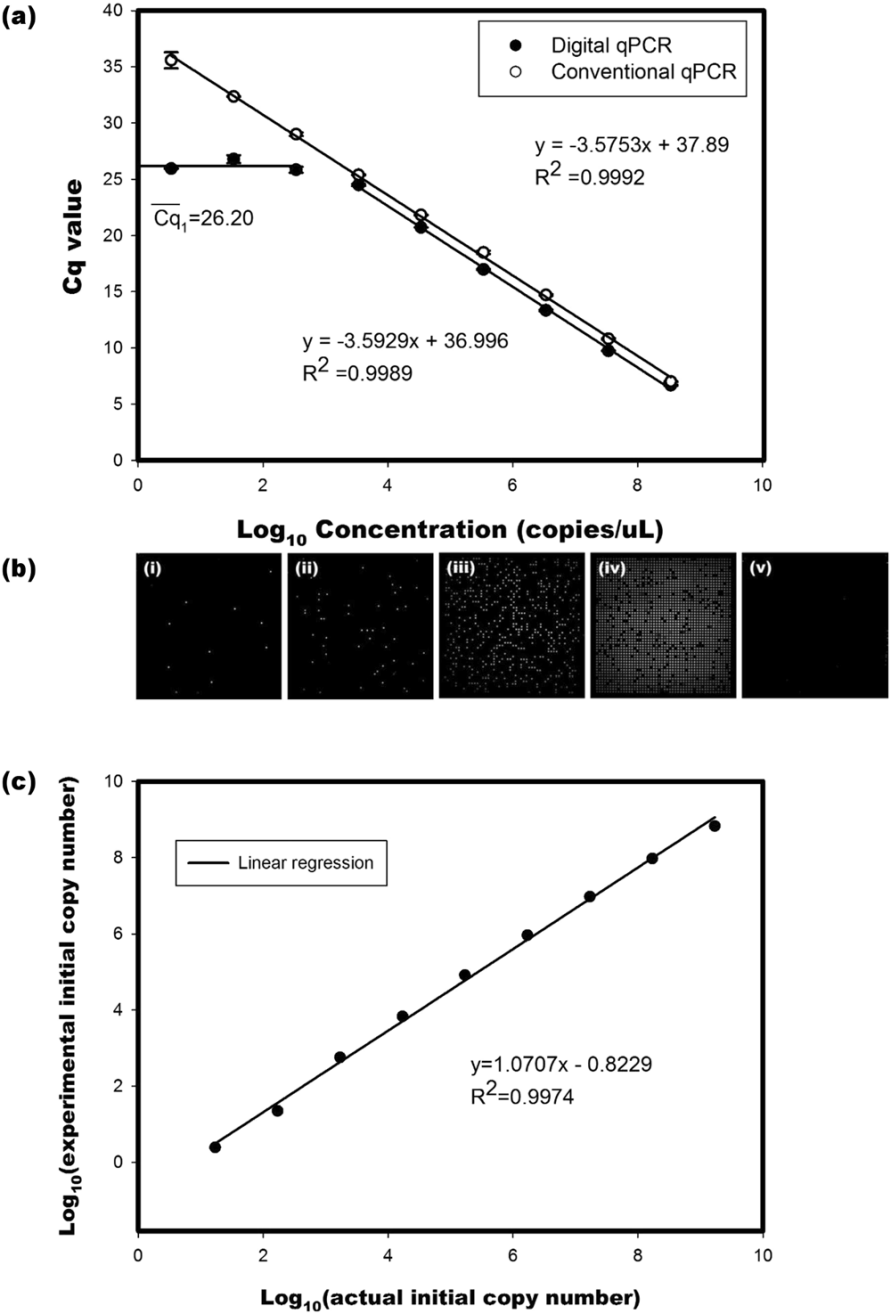
Serial dilution and qPCR from a 10-fold serial dilution of a pUC19 plasmid DNA standard (3.4 to 3.4 × 10^8^ copies copies/µL). (**a**) Quantification cycle (Cq) value against the logarithm of the starting pUC19 plasmid DNA concentrations. The efficiency levels associated with dqPCR and conventional qPCR were 89.81% (R^2^ = 0.9989) and 90.41% (R^2^ = 0.9992), respectively. The mean single copy Cq ($\overline{C{q}_{1}}$) was 26.20, which was derived from 3.4, 34.5, and 3.4 × 10^2^ copies/μL. (**b**) Fluorescence images of digital features at (i) 3.4, (ii) 34.5, (iii) 3.4 × 10^2^, and (iv) 3.4 × 10^3^ copies/μL, and (v) NTC. (**c**) Dynamic range of the PanelStation™ versus NanoDrop™. This figure shows that the experimental data adequately correlated with the actual initial copy number (estimated by NanoDrop™) (R^2^ = 0.9974).

A 10-fold serial dilution of a pUC19 plasmid DNA standard was performed, and the samples were processed through both dqPCR and qPCR. From 3.4 to 3.4 × 10^8^ copies/µL of the initial plasmid DNA template, the Cq values obtained from both dqPCR and qPCR were linear in relation to the initial concentration of pUC19 plasmid DNA. As illustrated in Fig. 1, as the initial concentration of the pUC19 plasmid DNA standard decreased, a linear slope in the five highest and a plateau region in the four lowest initial concentrations of pUC19 plasmid DNA were produced by the dqPCR assay. However, a linear slope in the whole initial concentration of pUC19 plasmid DNA was produced by the traditional qPCR assay. In the linear dynamic range, the amplification efficiency levels associated with dqPCR and conventional qPCR were 89.81% (R^2^ = 0.9989) and 90.41% (R^2^ = 0.9992), respectively.

Fluorescent images of the plateau region were generated in the microwell chip after 40 cycles of amplification, revealing that digital features appeared in the three lowest initial concentrations (3.4, 34.5, and 3.4 × 10^2^ copies/μL) of pUC19 plasmid DNA and a no template control (NTC) (Fig. 1b). As demonstrated by these images, the number of positive partitions decreased with the initial concentration of pUC19 plasmid DNA.

The Poisson equation was used to estimate the probability of one copy molecule in a positive partition. The estimation results revealed that the probabilities in the concentrations of 3.4, 34.5, and 3.4 × 10^2^ copies/μL were 100.0%, 100.0% and 98.5%, respectively (Table 1). Therefore, the positive partitions in the initial concentrations of 3.4, 34.5, and 3.4 × 10^2^ copies/μL almost contained one single target copy molecule, and the mean Cq value of a single copy $(\overline{\,C{q}_{1}})$ was 26.20.

**Table 1:** The digital characteristic of digital qPCR at low initial concentration.

#	Conc. of initial pUC19^a^ (copies/μL)	$\overline{C{q}_{1}}$	Positive Wells (mean) (k)	Fail Wells (mean)^b^	Total initial copies (ΣN^exp^)^c,d^	1 copy (%)^e^
(i)	3.4	26.32 ± 0.72	3.5	0	3.50	100.0
(ii)	34.5	26.58 ± 0.35	35	0	35.24	100.0
(iii)	3.4 × 10^2^	26.47 ± 0.37	454.5	0	501.52	98.5
(iv)	3.4 × 10^3^	25.36 ± 0.19	2301	0	6325.59	76.5
(v)	NTC	28.80 ± 5.08	1.67	0	1.67^f^	—

^a^The concentration was estimated from its A260/A280 ratio using the Nanodrop™.

^b^The PCR reagent was not introduced into wells, or the reactive wells with bubbles.

^c^Total initial copies in a 2,500 partitions microchip (n = 2,500).

^d^The total initial copies were estimated by ΣN^exp^(copies) = log(1 − k/n′)/log(1 − 1/n′), where n′ = 2500-Fail Wells– the number of primer dimer.

^e^The probability of the one copy in a positive well by ${\rm{P}}=\frac{{e}^{\lambda }{\lambda }^{x}}{x!}$, where *λ* = the total initial copies/(2500-fail wells); x is the actual number of copies in a partition, where x = 1.

^f^The number of primer dimer.

According to the number of positive partitions and the estimated initial molecules in a positive partition, the input molecule concentration (copies) at the digital level was measured by equation (3). Considering the effects of the primer dimer, the total amounts of initial molecules in partitions at the initial concentrations of 3.4, 34.5, 3.4 × 10^2^, and 3.4 × 10^3^ copies/μL were 2.44, 22.26, 567.66, and 6820.87 copies, respectively (Table 1).

Based on the resultant $\overline{C{q}_{1}}$ and efficiency, the experimental average of initial molecules in a positive partition for a higher initial concentration was estimated by equation (2) and correlated with the dynamic linear range of nine orders of magnitude in the dqPCR assay with a coefficient of 0.9974 (Fig. 1c).

#### Precision and repeatability

**Figure 2:**
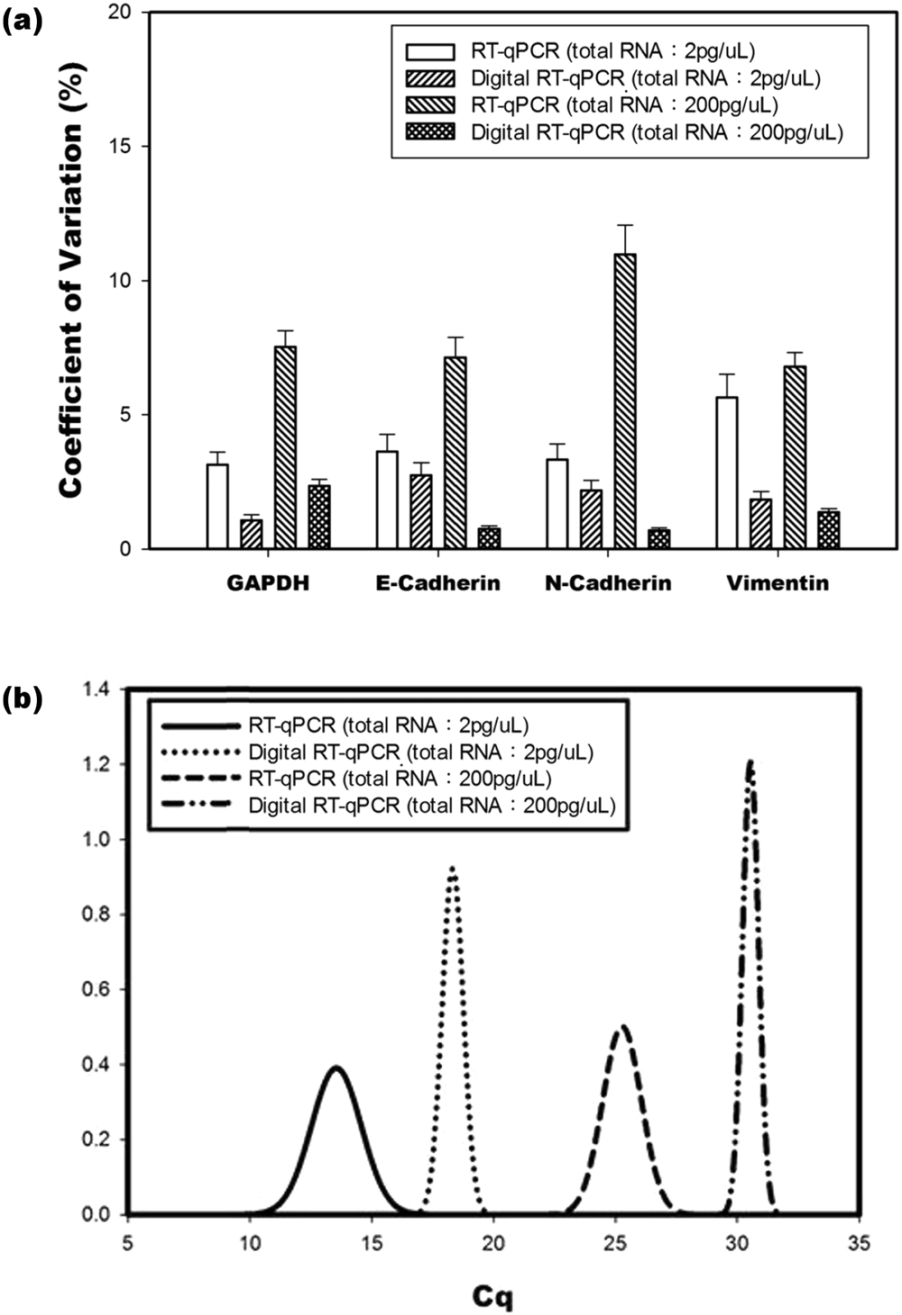
Precision and reproducibility comparisons of dqPCR and qPCR (**a**) on various gene (GAPDH, E-cadherin, N-cadherin, and vimentin) expression levels in low (2 pg/μL) and high (200 pg/μL) A549 total RNA concentrations. CV/SEM was determined with three replicates and repeated at least three times. The dqPCR and qPCR groups had a statistically significant difference in low (P < 0.05) and high (P < 0.001) concentrations. (**b**) Interexperimental variation of three replicates for dqPCR and conventional qPCR.

The coefficient of variation (CV) is an effective measure of the precision and reproducibility of dqPCR and a qPCR assay. The CV values of the interexperimental gene expression levels of A549 total RNA were measured at low (2 pg/μL) and high (200 pg/μL) initial concentrations using dqPCR and the conventional qPCR assay (Fig. 2). The average CV values for all samples in the low and high initial concentrations of A549 total RNA were 1.97% ± 0.70% and 1.30% ± 0.77%, respectively, for dqPCR; the CV values measured in the high and low concentrations were 3.94% ± 1.16% and 8.11% ± 1.93%, respectively, for qPCR. A t-test was performed to compare the CV values from the two methodologies. According to the comparison results, qPCR had more variability than dqPCR (6.024% ± 2.87% and 1.63 ± 0.82%, respectively); the differences reached statistical significance in both low and high initial concentrations of A549 total RNA (P < 0.05 and P < 0.001, respectively).

To analyse intraexperimental and interexperimental variations under each experimental condition, the mean and standard deviations from every experimental group of qPCR (n = 3) and dqPCR (n ≤ 1000) were generated using a normal distribution curve (bell curve) (Fig. 2b). For example, in GAPDH (loading template: 2 and 200 pg/ μL), in qPCR, the waveforms from the results of three replicates were dissimilar. A narrow bell curve width represents small intraexperimental variation in each single experiment; however, the variances between the groups were considerably large. If the bell curves do not overlap, it demonstrates that interexperimental variation is large (S5). For dqPCR, the curves were similar (though wide) and there was less variation in the width, meaning the results of replicates generated from the dqPCR system were stable (S5).

#### Inhibitor resistance

**Figure 3:**
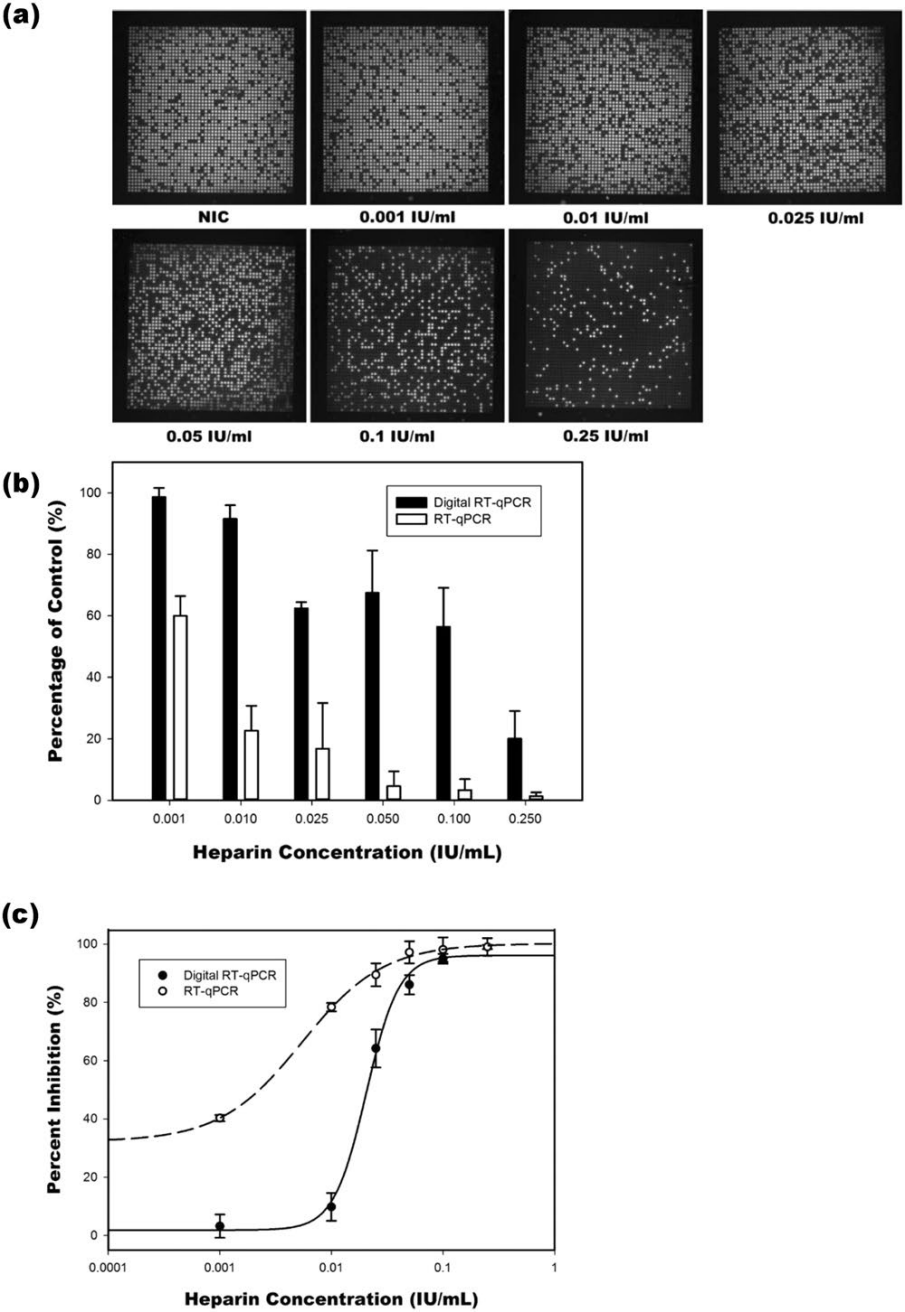
Heparin tolerance measurement using dqPCR and qPCR. (**a**) Fluorescence images of amplification with various heparin concentrations after 40 cycles for dqPCR. (**b**) Relative quantification of inhibited to NIC. (**c**) Inhibition curve of ddqPCR and qPCR. The IC50 values of dqPCR and qPCR were 0.02 and 0.002 IU/mL, respectively.

To assess the influence of the enzyme–inhibitor heparin on the reverse transcriptase PCR, various concentrations of heparin (0.001, 0.01, 0.025, 0.05, and 0.25 IU/mL) were mixed in a PCR reaction mixture containingA549 total RNA template, GAPDH primers, dNTP mixture, and Taq polymerase. The fluorescence image in Fig. 3a depicts the results of amplification with various heparin inhibitor concentrations in a dqPCR assay after 40 PCR cycles. The number of positive partitions decreased as the heparin concentration increased, indicating that certain enzymatic reactions in the partition were completely inhibited by heparin. An inverse relationship between the number of positive partitions and the concentration of the inhibitor heparin was observed.

Further analysis of the partially inhibited positive partitions in the presence of heparin revealed that heparin could still limit the number of amplification cycles. As indicated in Fig. 3b, qPCR and dqPCR exhibited different sensitivity levels in various concentrations of heparin. The percentage of control in the dqPCR assay was greater than that in the conventional qPCR at every heparin concentration (P < 0.001). At low inhibitor concentrations (0.001 and 0.01 IU/mL), the difference in the inhibitory effect of heparin was clearer. The percentage ratios derived for dqPCR were 98.61% ± 2.94% (at 0.001IU/mL) and 91.52% ± 4.42% (at 0.01IU/mL), whereas those derived for qPCR were 59.97% ± 6.38% (at 0.001IU/mL) and 22.67% ± 6.38% (at 0.01IU/mL). Even in the presence of the concentrated heparin inhibitor (0.25 IU/mL), the dqPCR assay showed superior tolerance to heparin (20.07% ± 8.98%), compared with the conventional qPCR (1.34% ± 1.24%).

Owing to the counted number of positive partitions and the relative inhibited initial molecules per positive partition, half maximal inhibitory concentration (IC50) values were estimated from the resulting inhibition curve (Fig. 3c). The IC50 value obtained for dqPCR (0.02 IU/mL) was approximately 10-fold higher than that obtained for qPCR (0.002 IU/mL) in the initial concentration of A549 total RNA (2 pg/μL); this indicates that the dqPCR assay showed significant tolerance to heparin.

### Cell distribution across the 2500 microwell chip

Because the probability of a cell settling into a well is random and independent, and because the distribution can be physically measured, a test can be conducted to determine whether a Poisson distribution can be used to mathematically model the system.

#### Cell distribution at various input numbers

**Figure 4:**
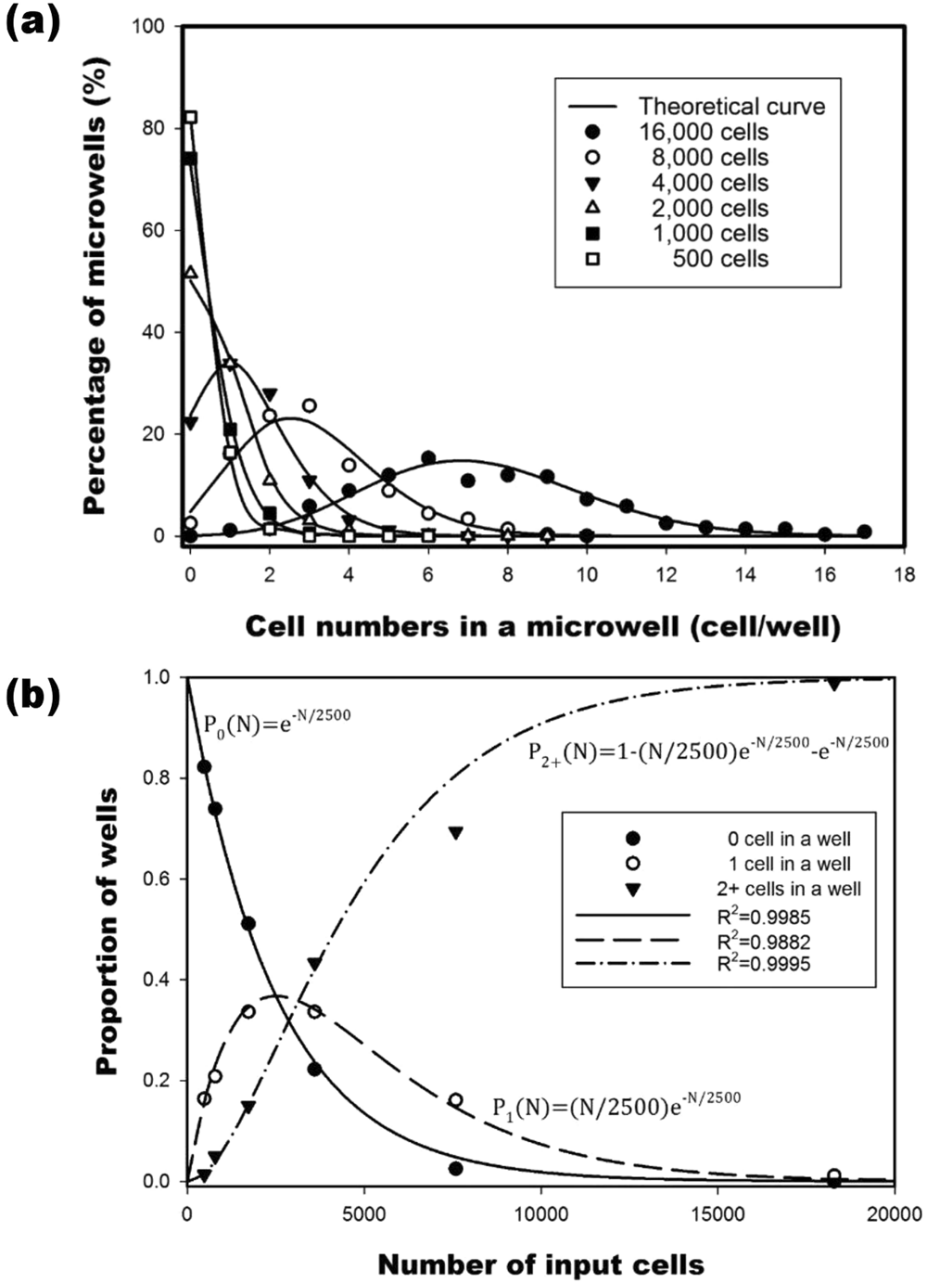
Different numbers of cells were spread in a 2500-microwell chip. (**a**) Distribution of the number of cells in each well was compared with the theoretical Poisson distribution. (**b**) Theoretical fitting of the proportion of zero (closed circle), one (open circle), and two (or more) cells (inverted triangle) in a well at various numbers of input cells. Equation for theoretical fitting: P_0_(N) = e^−N/2500^, R^2^ = 0.9985; P_1_(N) = (N/2500)e^−N/2500^, R^2^ = 0.9882; P_2+_(N) = 1 − (N/2500)e^−N/2500^ − e^−N/2500^, R^2^ = 0.9995.

Theoretical results were compared with experimental data, revealing that the distributions of the cells in each well at each cell serial dilution were fitted with the Poisson distribution (Fig. 4a). Nonlinear regression analysis was performed using the data points from the cell serial dilution experiment against equations (9), (10) and (11). The R-squared values from the nonlinear regression were as follows: 0.9985 for 0 cells, 0.9882 for 1 cell, and 0.9995 for multiple cells (Fig. 4b). As the input cells reached 500, the probability of one cell (single cell) being in a positive well was 90.33%. The proportion of single cells increased as the number of input cells decreased.

### Epithelial-mesenchymal gene expression profiling in single cells

**Figure 5:**
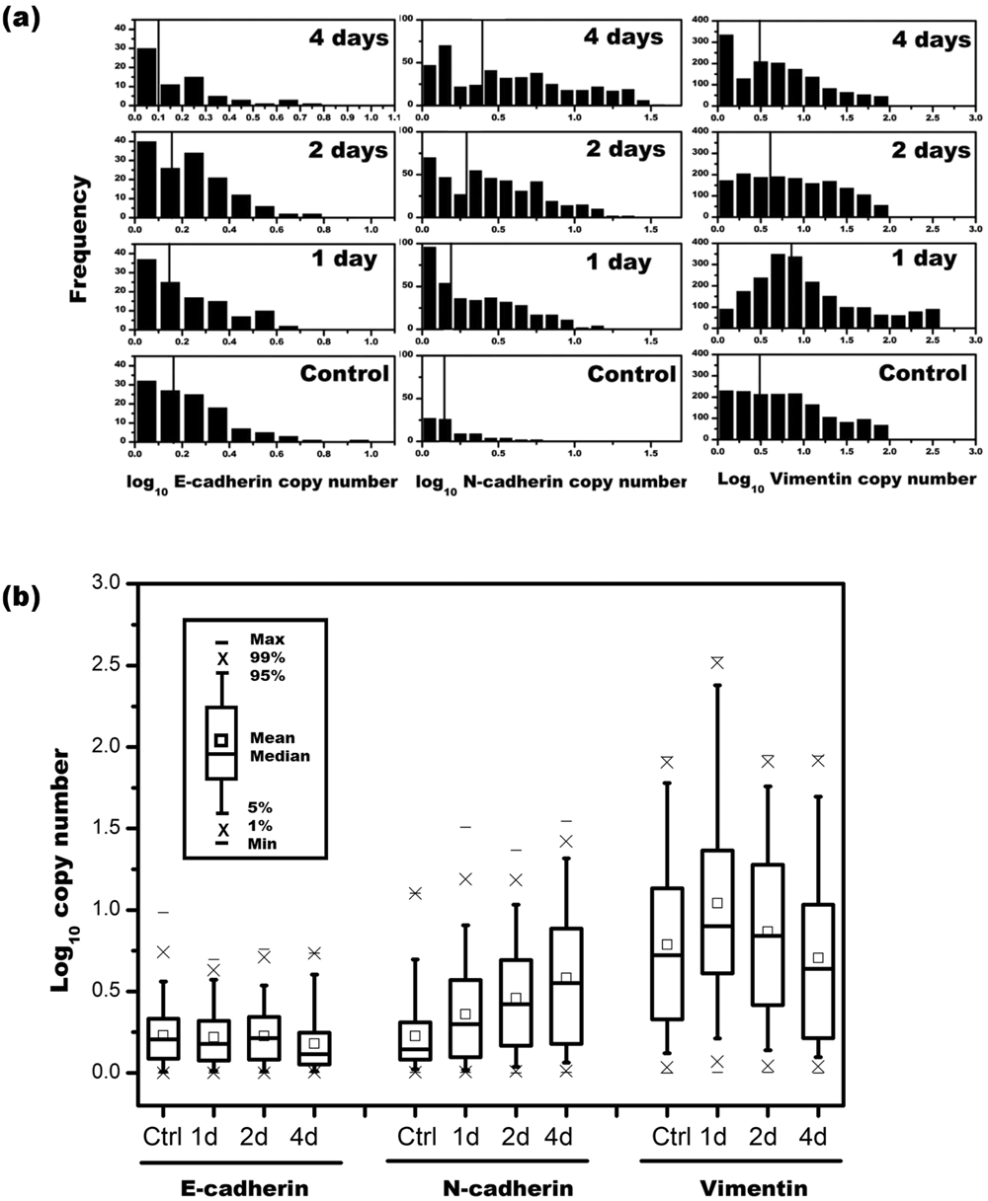
Gene expression results of a single cell treated with TGFβ1 for various days. (**a**) Histogram binned by log of calculated copy number for (left) E-cadherin, (middle) N-cadherin, and (right) vimentin, at various time stamps. The vertical lines show the geometric mean of each group. (**b**) Box–whisker plot of the log of the copy number for the various times and treatment groups.

Figure 5a shows histograms of the logarithm of E-cadherin, N-cadherin, and vimentin expression in A549 cells treated with 5 ng/mL of TGFβ1 at 1 day, 2 days, and 4 days, as well as in a no treatment control. When the histograms were tested for normality and lognormality using the one-sample Kolmogorov–Smirnov (K–S) test, the results were strongly negative for all groups (S6), indicating that the expression levels of these genes had an asymmetric distribution. Therefore, a two-sample K–S test was used to detect differential gene expression at different time points (S7). According to the K–S test of significance comparing the expression levels of the different genes between the various groups, N-cadherin and vimentin clearly exhibited statistical significance in all the compared groups, whereas E-cadherin showed little to no signs of statistical significance. As revealed by the results of the geometric mean at each time point (Fig. 5a) and the box–whisker plot (Fig. 5b), the expression of N-cadherin increased with the TGFβ1 treatment time, and the expression of vimentin first increased and then decreased with the TGFβ1 treatment time. However, the expression of E-cadherin did not exhibit any obvious trend in the 4 days.

## Discussion

The PanelChip™ Analysis System based on dqPCR is a powerful tool for measuring gene expression in the presence of interference and has the potential for single-cell application. The sample and qPCR reagent mixture were diluted and partitioned into numerous individual small-volume reactions that could effectively improve the detection limit, increase precision, and raise the tolerance level to the PCR inhibitor.

**Figure 6:**
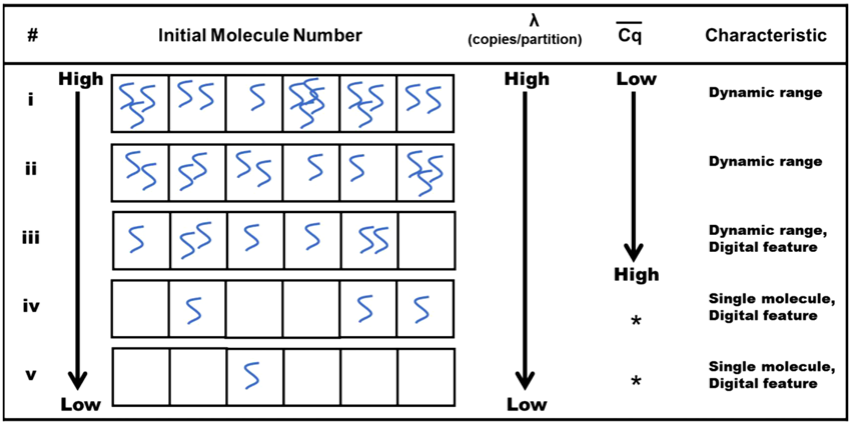
Various features of the different molecules were dispersed into individual partitions. The mean number of copies per partition (λ) decreased as the amount of initial molecules decreased (i-v). The mean Cq values rose as the initial molecules number decreased at dynamic range (i-iii). The digital features (partitions contain with or without molecules) appeared below a certain amount of initial molecules (iii-v). The mean Cq values were constant at extremely low amounts of initial molecules (asterisk). The Cq values of one single copy are generated at extremely low amounts of initial molecules; therefore, the mean Cq values (defined as $\overline{C{q}_{1}}$) were theoretically the same and constant (iv and v).

A serial dilution of the template molecules was partitioned into a microwell array chip containing 2500 individual reaction partitions with various behaviours based on Poisson statistics^17^. The mean copies per partition (λ) change were determined to be dependent on the amount of initial sample molecules (Fig. 6i–v). When the initial target template molecules were diluted to a certain amount and distributed into the individual partitions, the partitions containing no target template molecule were observed; this was called ‘the digital feature’ (with or without a target template) (Fig. 6iii–v). In the digital feature range, the number of positive partitions (k) can be used to estimate the true number of initial target template molecules according to the following equation: λ (mean copies per partition) = −ln(1 − k/n), where **n** is the number of partitions^22^. Lower Cq values indicate amplification with a higher initial concentration of target templates in a partition, and higher Cq values indicate a lower initial concentration of target templates. Consequently, reducing the amount of initial target templates could increase the average Cq values per partition, as demonstrated in Fig. 6i–iii.

The CV levels of the Cq values were compared between the two methodologies. A lower CV suggests a higher degree of consistency among results, which further suggests a higher degree of precision. Our results confirm that for all initial concentrations of template RNA, the CV values of the data from the novel dqPCR were all lower than those of the data from the conventional qPCR. A possible reason for this finding is that the dimensions of the reaction microwells and the mechanized distribution of reagents allowed for a better controlled PCR environment. Additionally, the sheer number of reaction wells in the dqPCR assay used in this study mathematically drove down the CV by increasing the probability of wells containing a relatively even number of RNA copies through Poisson statistics. Specifically, the possibility of wells containing a particularly high number of RNA copies was eliminated. A PCR solution is divided into three equal portions and hundreds (or thousands) of aliquots, meaning standard deviation values can be quite different. Samples are divided into three portions, and the difference between portions is theoretically quite small. When the molecules of a sample are dispersed into hundreds (or thousands) of parts, each portion is affected by a probability distribution and each aliquot as well, thus increasing standard deviation. The dqPCR results from the normal distribution curve and the CV graph indicate that the interexperimental variation of dqPCR was smaller than that of qPCR. A possible reason is that dividing the sample into thousands of portions can result in an average value close to the true value of the results after the PCR process. Therefore, compared with qPCR, the results from dqPCR were determined to have high reproducibility and high stability.

Partitioning samples improves tolerance to inhibitors. According to our data, the dqPCR assay was more tolerant to heparin than the conventional qPCR assay, suggesting that a partitioned PCR reaction should reduce susceptibility to PCR inhibitors. In a PCR reaction solution containing a certain proportion of target templates and inhibitory substances, reducing the concentration of the entire reaction would not change the proportion. Partitioning a sample into parts can effectively segregate the target nucleic acid and PCR inhibitor before the start of the reaction. Hence, the ratio of the target nucleic acid and inhibitor decreases in the partition to mitigate the influence of the inhibitor on the enzymatic reaction. Because the PCR method enables logarithmic amplification, a slight difference of reaction in every amplification step would be accumulated and result in a reduced Cq value^25^. The dqPCR assay has the potential to reduce the negative influence of inhibitors, whereas trace amounts of inhibitors can significantly affect results when using a conventional qPCR system. However, although the experimental data show that the dqPCR system can effectively reduce the inhibitory effect of heparin on the PCR process, the partition strategy co effectively reduce the effect of other kinds of inhibitors. For example, one PCR inhibitor, ethylenediaminetetraacetic acid (EDTA), can chelate magnesium ions. The presence of EDTA-chelated magnesium ions in a PCR solution leads to the absence of adequate free magnesium, thus reducing the activity of Taq DNA polymerase, an essential enzyme in PCR. In this case, partitioning the reaction solution into parts reduces the proportion of magnesium ions and enzymes, which hinders the PCR process^24^. A positive of such behaviour is that the dqPCR assay may be useful in the study of inhibited PCR processes by distinguishing different types of inhibition mechanisms.

Cells were spread across a chip to demonstrate that the actual distribution of the cells conformed to the Poisson distribution, and that the preceding equations could be used to model the system. Finding such a result is significant as we can then make mathematical assumptions about the cellular distribution over the 2500 microwell chip with greater confidence.

According to the K–S test of significance comparing the expression levels of the different proteins between the two methods, N-cadherin and vimentin clearly exhibited statistical significance in all the compared groups, whereas E-cadherin showed little to no signs of statistical significance. Although E-cadherin did not exhibit significant changes in expression in A549 lung cancer cells after the application of TGFβ1, this was assumed to be due to the length of the time stamps used to measure their expression. The expression of E-cadherin may have changed significantly in the first few minutes to hours of exposure to the drug. Apart from E-cadherin, PanelChip™ Analysis System enabled the detection of changes in the mRNA expression levels of N-cadherin and vimentin in A549 cells in the days following TGFβ1 application.

## Methods

### Reference materials

#### Cell Sample (A549 Preparation) and Total RNA Isolation

A549 lung carcinoma cells (Bioresource Collection and Research Centre, Taiwan) were cultured on 6-cm GeneDirex culture dishes in Dulbecco’s Modified Eagle Medium (DMEM; Gibco, Life Technologies, Taiwan) at 4 × 10^5^ cells per 5 mL DMEM. The medium was supplemented with 10% fetal bovine serum and 100 u/mL penicillin and 100 ng/mL streptomycin at 37 °C and over 5% CO_2_. Total RNA was extracted and purified from the A549 lung carcinoma cells by using Trizol® RNA isolation reagents (Thermo Fisher Scientific, Taiwan), according to the manufacturer’s instructions. The total RNA purity and concentration were estimated from the corresponding A260/A280 ratio by using Infinite 200 Pro (Life Sciences, Taiwan). A549 total RNA was stored at −80 °C.

#### pUC19 Standard

The pUC19 standard and primer pairs of vimentin, E-cadherin, N-cadherin, and GAPDH were purchased from Protech Technology Enterprise Co (Taipei, Taiwan) (S2). The pUC19 plasmid DNA standard was quantitated using Nanodrop™ (Thermo Fisher Scientific, Taiwan) and converted into the DNA copy number used in the calculations in this study. All samples were diluted to appropriate concentrations with nuclease-free water and stored at −80 °C.

### Amplification methods

#### RT-qPCR and qPCR

One-step RT-qPCR reactions were performed in 96-well plates using QuarkBio™ 1-Step RT-qPCR System (Quark Biosciences, Inc.), according to the manufacturer’s instructions. The reaction mixture contained 2 µL of template DNA (or total RNA in RT-qPCR), 10 µL of 2 × QuarkBio qPCR master mix (Quark Biosciences, Inc.), 0.4 µL of 50 × QuarkBio™ RT Mix (Quark Biosciences, Inc.), 2 µL of 10 µM primer pair, and nuclease-free water, resulting in a reaction volume of 20 µL. All reactions were performed in triplicate. Plate cycling was conducted with the CFX Connect™ Real-Time PCR Detection System (BioRad) with the following parameters: 42 °C for 20 min (RT-qPCR only), 95 °C for 2 minutes, followed by 40 cycles at 95 °C for 5 seconds and at 60 °C for 30 seconds.

#### dqPCR

The qPCR mixture (50 µL) was introduced into the PanelChip™ Analysis System (Quark Biosciences) on a microwell array chip^29^, according to the manufacturer’s instructions (S1). The chips were fabricated with a high-transmittance material, polycarbonate, which was coated with a thin layer of 0.1% Triton X-100 (Sigma-Aldrich). Enclosed in the 22.5 mm × 22.5 mm chip were 2500 wells with a volume of 20 nL per partition, totalling 50 µL of volume per chip (S3). In the process of qPCR cycling, the fluorescence intensity of each well was detected and collected with a charge-coupled device detector installed onto PanelChip™ Analysis System. The reagents were the same as the qPCR assay. The conditions of thermal cycle: 42 °C for 20 min (RT-qPCR only), 95 °C for 3 minutes, followed by 40 cycles at 95 °C for 36 seconds and at 60 °C for 72 seconds.

### Linearity and sensitivity of dqPCR and qPCR platforms

For estimating the sensitivity of the measure of absolute copy number in dqPCR and qPCR, a serial dilution of pUC19 plasmid DNA was used. The amplification efficiency (E) was determined on the dqPCR and qPCR platforms by using triplicates of a 10-fold dilution series of pUC19 plasmid DNA (from 3.4 to 3.4 × 10^8^ copies/µL of solution) as a template. All Cq values of the dilution series were measured in triplicate and then plotted on a graph. The resultant slope was used for the calculation of efficiency according to equation (1).

1 $$E={10}^{-1/slope}$$

### Precision and repeatability

To measure the repeatability of the measure of gene expression, high (200 pg/µL) and low (2 pg/µL) initial template concentrations were measured by dqPCR and qPCR. The primers of four genes known to be expressed in A549 cells (GAPDH, E-cadherin, N-cadherin, and vimentin) were included in the reaction mixture. The measurement of Cq for the four genes at each template concentration was repeated three times. The CV of each group was then calculated as a measure of variability.

### Inhibitor resistance

Heparin (Sigma-Aldrich) was added to the qPCR reaction mixture at various concentrations and subjected to dqPCR and qPCR to evaluate the influence of the inhibitor on the resultant Cq value from the two quantification methods. The reaction mixtures each contained 1 µL of 2 pg/µL total RNA, 2 µL of 10 nM GAPDH primer pair, 10 µL of 2 × GoTag® qPCR Master Mix, 0.4 µL of 50 × GoScript™ RT Mix, and 1 µL of heparin at various concentrations (0, 0.02, 0.2, 0.5, 1.0, and 5.0 IU/mL). The measurement of GAPDH at each concentration of heparin for dqPCR and qPCR was repeated three times.

### Single-cell methods

#### Cellular distribution across chip

Various amounts (approximately 16,000, 8000, 4000, 2000, 1000, 500 cells/50 μL) of cells in suspension of phosphate buffered saline were spread onto the 2500 wells of the chip that were not coated with Triton. The chip was viewed under an optical microscope to take cell counts of each well and to count the number of wells alongside the numbers of individual cells inside.

#### Epithelial–mesenchymal transformation

For the epithelial–mesenchymal transition experimental group, the A549 cells were treated with 5 ng/mL of TGFβ1 (Sigma-Aldrich) in DMEM, whereas the control group remained untreated. The cells were harvested using trypsin and diluted down to approximately 500 cells/60 µL to be spread onto the chip. The cells in the TGFβ1 group were treated with TGFβ1 for 1 day, 2 days, and 4 days. RT-qPCR was performed for each of those groups for the mRNA of E-cadherin, N-cadherin, and vimentin and treated with their respective RNA primers.

### Data analysis

#### Dynamic range of dqPCR

In dqPCR, the initial sample of the DNA template solution is separated into numerous partitions such that in the case of an extremely diluted template solution, most partitions would contain either 1 or 0 copies of the template. Therefore, it is possible to obtain the Cq value of a single copy of the target DNA (Cq_1_), the theoretical maximum Cq value. Subsequently, the number of DNA templates can be estimated given any Cq value through the following ratio:

2 $$N/{N}_{1}={(1+E)}^{(Cq-C{q}_{1})}$$

where *N* is the estimated number of initial template copies, *N*_1_ is the number of template copies when there is one copy (simply equalling 1), *E* is the amplification efficiency at which a particular machine operates (with 1.0 meaning 100% efficiency, or that all copies have exactly doubled at each cycle), *Cq* is the value obtained from each particular experiment, and *Cq*_1_ is the Cq value obtained from when there is only one copy of the template. Additionally, the number of copies present can be estimated using equation (3) used for dqPCR^26^ because the sample is separated into partitions in a similar fashion:

3 $$N=\frac{\mathrm{log}(1-k/n)}{\mathrm{log}(1-1/n)}$$

where *N* is the estimated number of initial template copies, *k* is the number of partitions that contained nucleic acids after amplification (i.e. positive partitions), and *n* is the number of partitions that the sample was separated into (2500).

*Evaluation of the inhibitory influence of heparin*. To create a measure of the inhibitory influence of heparin, that the parameter being affected is assumed to be the PCR amplification efficiency *E* such that an inhibitory parameter *E′* can be included in the calculations. Then, the PCR process under the influence of heparin is assumed to reach a threshold *K*, as presented in equation (4):

4 $$K=N{(1+E+{E}^{\text{'}})}^{Cq\text{'}}$$

where *K* is the threshold that an inhibited PCR reaction reaches, *N* is the initial number of nucleic acid template copies, *E* is the efficiency of the PCR machine, *E′* is the inhibitory parameter, and *Cq′* is the Cq value as a result of the influence of the inhibitor heparin. Then, this equation is rewritten into an equation that estimates the number of initial template copies without consideration of inhibition in equation (5):

5 $$K=N\text{'}{(1+E)}^{Cq\text{'}}$$

where *N* is the estimated initial copy number without consideration of inhibition, *E* is the efficiency of the PCR machine, and *Cq* is the raw Cq value obtained from a particular run of the PCR assay. Through the use of a ‘no inhibitor control’ (NIC) sample, the degree to which heparin inhibits the qPCR and dqPCR processes was evaluated in this study in comparison with uninhibited conditions by using equation (6):

6 $$\frac{N^{\prime} }{{N}_{NIC}}\times 100{\rm{ \% }}={(1+E)}^{(CqNIC-Cq^{\prime} )}\times 100{\rm{ \% }}$$

where *N′* is the estimated initial copy number obtained from the inhibited sample, *N*_*NIC*_ is the estimated initial copy number of the NIC, *E* is the calculated efficiency of the PCR machine, *Cq*_*NIC*_ is the calculated Cq value of the NIC, and *Cq* is the calculated Cq value of the inhibited sample.

#### Cellular distribution in a chip

Under ideal conditions, where the cells’ motion and position in solution are random and independent of one another, the number of wells that contain one or more cell is related to the actual number of input cells, according to equation (3)^30^:

7 $${\rm{X}}=\frac{{\rm{l}}{\rm{o}}{\rm{g}}(1-\frac{k^{\prime} }{2500})}{{\rm{l}}{\rm{o}}{\rm{g}}(1-\frac{1}{2500})}$$

where *k′* is the number of reactive wells and *X* is the total input number.

Additionally, the number of cells falling into each compartment should follow the Poisson distribution:

8 $${\rm{P}}({\rm{k}}^{\prime} ,{\rm{N}})=\frac{{(\frac{N}{2500})}^{{k}^{^{\prime} }}{e}^{-N/2500}}{k^{\prime} !}$$

where *P* (k′, N) is the proportion of wells containing k′ number of cells and *N* is the total number of input cells.

The following three equations are based on the Poisson distribution in equation (7) and are the probabilistic model functions of the proportion of wells containing 0, 1 and 2 + cells:Zero cell:

9 $${{\rm{P}}}_{0}({\rm{N}})=\frac{{(\frac{N}{2500})}^{0}{e}^{-N/2500}}{0!}$$

One cell:

10 $${{\rm{P}}}_{1}({\rm{N}})=\,\frac{{(\frac{N}{2500})}^{1}{e}^{-N/2500}}{1!}$$

Two or more cells:

11 $${{\rm{P}}}_{2+}({\rm{N}})=1-{{\rm{P}}}_{1}({\rm{N}})-\,{{\rm{P}}}_{0}({\rm{N}})$$

where *P* is the proportion and *X* is the total number of cells inputted.

A nonlinear regression analysis was performed to compare the fit of our functions with the actual proportion of cells landing in various amounts in the wells.

### Single-cell gene expression profiling

The geometric means^31^ of the Cq values from the reactive wells were determined with the assumption that each of the wells contained a single cell^31,32^. The resulting mean Cq value for a single GAPDH reference gene, Cq_1_, from the A549 lysate was determined (S4) and applied to the following equation (12) that calculates the initial number of target genes in a cell.

12 $${\rm{A}}={2}^{Cq1-Cq}$$

where *A* is the initial number of mRNA copies in a cell, *Cq*_1_ is the Cq value that results from a single GAPDH reference gene, and *Cq* is the Cq value of the well in question.

The resulting data were processed through equation (12) to obtain the number of target genes of a single cell in a well. The target genes in a single cell were compiled into histograms using OriginPro, and the geometric means of the copy numbers were derived. A one-sample K–S test was performed to determine the fit of each copy number histogram to a normal and lognormal distribution using SigmaPlot 12.0. Two-sample pairwise K–S tests were then used for determining statistical significance.

## Electronic supplementary material

supplemental data
