# Supplementary material for: Evaluation of digital real-time PCR assay as a molecular diagnostic tool for single-cell analysis
Source: Sci Rep. 2018 Feb 21;8:3432. doi: 10.1038/s41598-018-21041-5 (PMC5821883; doi:10.1038/s41598-018-21041-5)
Supplement: Supplementary file 1 — supplemental data [file 41598_2018_21041_MOESM1_ESM.pdf]

Evaluation of digital real-time PCR assay as a molecular diagnostic tool for single-cell analysis

Chia-Hao Chang[1], Daxen Mau-Hsu[2], Ke-Cheng Chen[2,3], Cheng-Wey Wei[4], Chiung-Ying Chiu[4], Tai-Horng Young[1,2,*]

[1]Institute of Polymer Science and Engineering, Taipei, 106, Taiwan

[2]Institute of Biomedical Engineering, Taipei, 100, Taiwan

[3]National Taiwan University Hospital, 100, Taiwan

[4]Quark Biosciences, Inc., Hsinchu County, 302, Taiwan

* thyoung@ntu.edu.tw

Supplemental data

S1. To load the samples onto microwell array chip (PanelChip™) and set up onto PanelChip™ Analysis System.

The PCR mixture contained template DNA (or total RNA in RT-qPCR or cells), 2X GoTaq® qPCR Master Mix, 50X GoScript™ RT Mix, primer pair, and nuclease-free water, resulting in a reaction volume of 60 μL. The PCR mixture was then applied onto microwell array chip by a glass slide (step a).

Each chip is sealed in mineral oil and then flipped upside down to prevent the evaporation of the liquid solvent (step b). The chips are set up onto the PanelStation™ for the RT-qPCR protocol. The microwell array chips go through a thermocycling protocol which is initiated by 20 minutes at 42℃ to generate cDNA using reverse transcriptase followed by 40 cycles of PCR (3 minutes of 95℃ activation, 72 seconds of 60℃ extension and 36 seconds of 95℃ denaturation) (step c). At the end of each cycle, the CCD detector in the PanelStaion™ records the emission from BRYT Green®, which can be detected at 530 nm; the cycle number at which the emission from BRYT Green® reaches threshold is recorded as the Cq value.

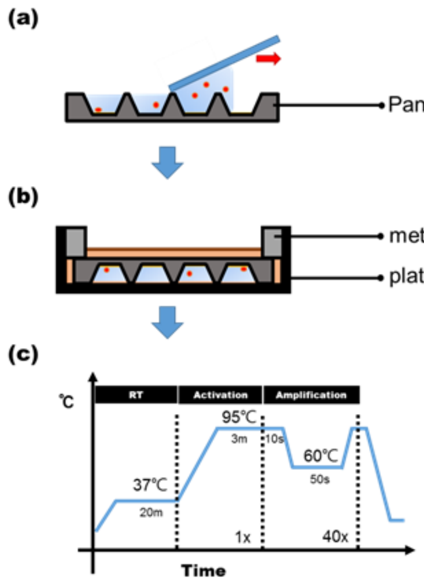

Schematic of the microwell array chip plating process; (a) application of PCR mixture across microwell array chip via glass slide; (b) immersion of microwell array chip into mineral oil; and (c) the polymerase chain reaction process.

S2. The pUC19 plasmid DNA standard sequence and list of primers information for qPCR

pU19 plasmid DNA:

ccggatcaagagctaccaactctttttccgaaggtaactggcttcagcagagcgcagataccaaatactgttcttctagtgtagccgtagttaggccaccacttcaagaactctgtagcaccgcctacatacctcgctctgctaatcctgttaccagtggctgctgccagtggcgataagtcgtgtcttaccgggttggact

Symbol	Forward primer	Reverse primer	Amplicon size (bp)
pUC19	AGTCCAACCCGGTAAGACAC	CCGGATCAAGAGCTACCAA	202
E-cadherin[1]	TGCCCAGAAAATGAAAAAGG	GTGTATGTGGCAATGCGTTC	200
N-cadherin[1]	ACAGTGGCCACCTACAAAGG	CCGAGATGGGGTTGATAATG	201
Vimentin[1]	GAGAACTTTGCCGTTGAAGC	GCTTCCTGTAGGTGGCAATC	163
GAPDH[2]	AGTAGAGGCAGGGATGATGTTC	CTTTGGTATCGTGGAAGGACTC	133

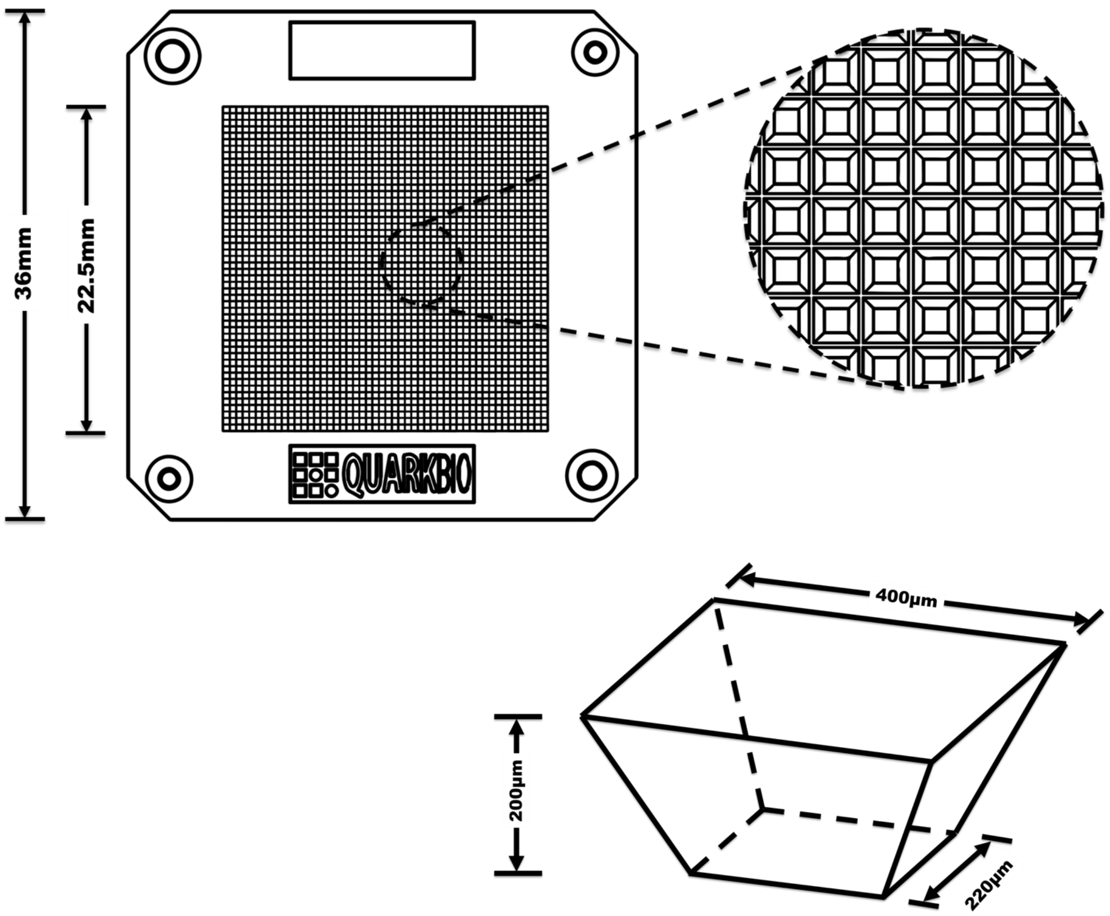

S3. Schematic diagram of microwell array chip. The microwell array chip was composited by 2500 partition wells in the 22.5mm by 22.5mm, each of them with a volume of 20nL.

(A)

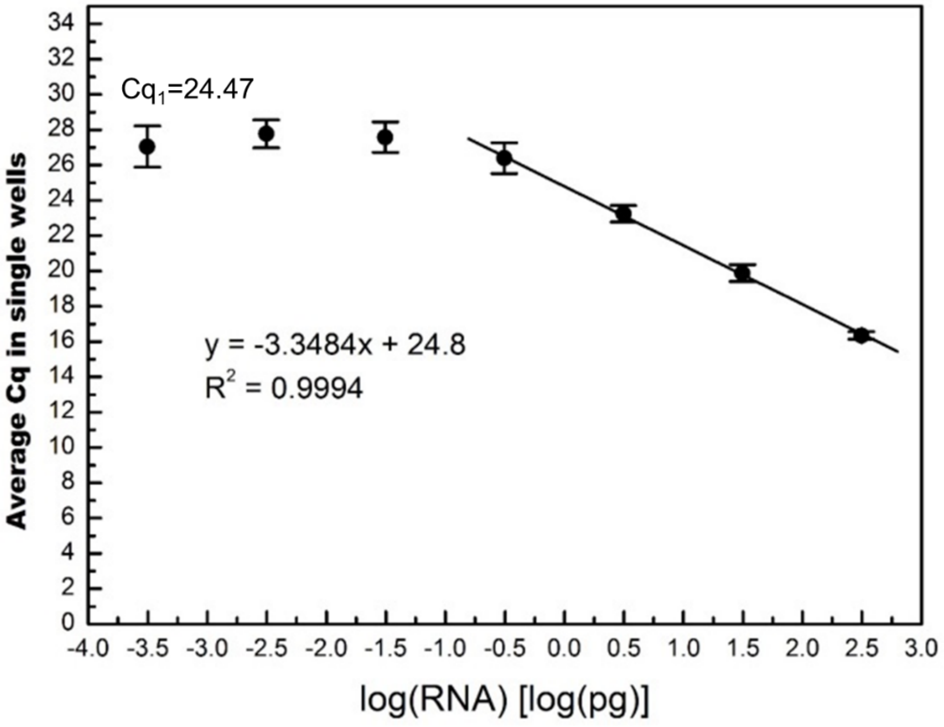

(B)

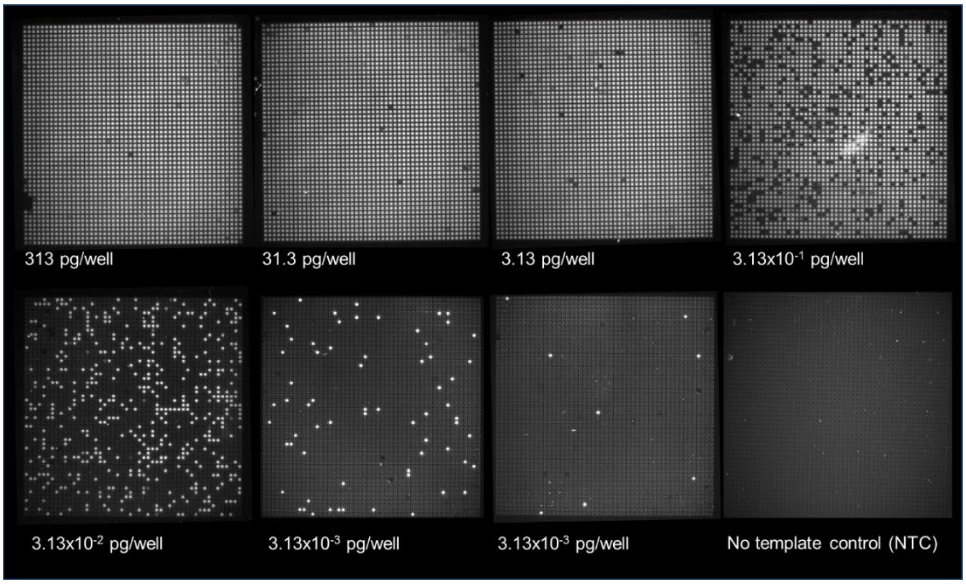

S4. Serial dilution of A549 cell lysate. (A) The 10x serial dilution of RNA lysate ranging from 15.65ng/μl to 1.56x10^{-5} ng/μl was performed. Using the data points from the lowest of dilutions of GAPDH mRNA (1.56 x10^{-4} and -05), the mean single copy Cq for A549 cell lysate was 27.47. (B) The fluoresce images of serial dilution of cell lysate.

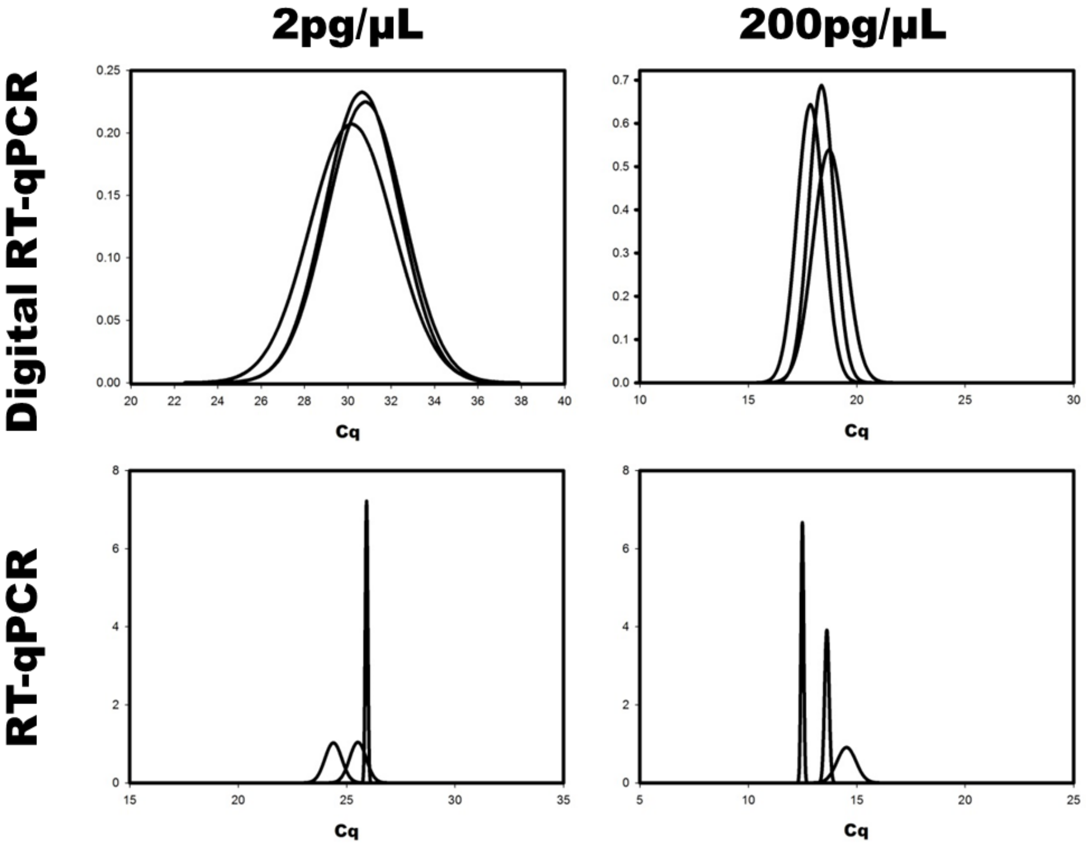

S5. Normal distribution curve of three replicates Cq results using conventional RT-qPCR and digital RT-qPCR at two concentrations of template for GAPDH. The reverse-transcription quantitative polymerase chain reaction was accomplished with digital RT-qPCR (above) and conventional RT-qPCR (below) when A549 total RNA are 2 pg/μL (left) and 200 pg/μL (right), respectively.

S6. One-sample Kolmogorov-Smirnov statistical tests to determine the normality of each copy number histogram to a normal and lognormal distribution.

Group	Gene	n*	Arithmetic mean	SD	Geometric mean	log_{10} of geometric mean	KS test**	KS test*** (log_{10})
TGFβ1 Day 1	E-cadherin	113	1.812	0.854	1.657	0.128	-	-
	N-cadherin	370	3.009	2.985	2.288	0.215	-	-
	Vimentin	2055	37.086	0.652	14.161	0.836	-	-
TGFβ1 Day 2	E-cadherin	143	1.835	0.839	1.689	0.134	-	-
	N-cadherin	423	3.821	3.337	2.872	0.304	-	-
	Vimentin	1567	14.661	18.233	7.354	0.658	-	-
TGFβ1 Day 4	E-cadherin	69	1.670	0.912	1.514	0.099	-	-
	N-cadherin	433	6.046	6.308	3.823	0.394	-	-
	Vimentin	1434	10.739	16.430	5.083	0.498	-	-
Control	E-cadherin	119	1.881	1.138	1.700	0.1408	-	-
	N-cadherin	85	1.996	1.753	1.681	0.143	-	-
	Vimentin	1617	13.037	18.517	6.139	1.390	-	-

* number of positive wells
** and *** “minus” means that experimental results did not fitted with standard normal distribution

S7. Pairwise Kolmogorov-Smirnov statistical tests for significance between the various days between each mRNA group.

E-cadherin	control	1day	2days	4days
control				
1day	-			
2days	+	-		
4days	-	-	-	

N-cadherin	control	1day	2days	4days
control				
1day	+			
2days	+	+		
4days	++	+	++	

Vimentin	control	1day	2days	4days
control				
1day	++			
2days	++	++		
4days	++	++	++	

- p>0.01
+ p<0.01
++ p<0.001.

Supplemental References

1. Theys, J. *et al.* E-Cadherin loss associated with EMT promotes radioresistance in human tumor cells. *Radiotherapy and oncology* **99**, 392-397 (2011).
2. Watanabe, T. *et al.* Comparison of lung cancer cell lines representing four histopathological subtypes with gene expression profiling using quantitative real-time PCR. *Cancer cell international* **10**, 1 (2010).
